# Epigenetic silencing of LncRNA LINC00261 promotes c-myc-mediated aerobic glycolysis by regulating miR-222-3p/HIPK2/ERK axis and sequestering IGF2BP1

**DOI:** 10.1038/s41388-020-01525-3

**Published:** 2020-10-29

**Authors:** Shuyu Zhai, Zhiwei Xu, Junjie Xie, Jun Zhang, Xinjing Wang, Chenghong Peng, Hongwei Li, Hao Chen, Baiyong Shen, Xiaxing Deng

**Affiliations:** 1 Department of General Surgery, Pancreatic Disease Center, Research Institute of Pancreatic Diseases, Ruijin Hospital, Shanghai Jiaotong University School of Medicine, Shanghai, China; 2 Research Institute of Pancreatic Diseases, Shanghai Jiaotong University School of Medicine, Shanghai, China; 3 State Key Laboratory of Oncogenes and Related Genes, Institute of Translational Medicine, Shanghai Jiaotong University, Shanghai, China

**Keywords:** Cancer metabolism, Pancreatic cancer

## Abstract

Long noncoding RNAs have been identified as key regulators in the progression of various cancers. LINC00261 has been reported as a tumor suppressor in multiple cancers. However, its function and underlying mechanisms in pancreatic cancer remain largely unclear. Quantitative real-time PCR was performed to detect RNA expression. In situ hybridization was used to discover the subcellular location. The direct binding of LINC00261 to miR-222-3p was verified using a dual-luciferase reporter assay and RNA immunoprecipitation. LINC00261-binding proteins were detected using an RNA pulldown assay. LINC00261 was downregulated in pancreatic cancer tissues and cell lines. Its reduced expression was correlated with advanced pathological stage and poor prognosis. Forced expression of LINC00261 suppressed pancreatic cancer glycolysis and proliferation and induced cell cycle arrest and apoptosis. Mechanistically, downregulation of LINC00261 was caused by hypermethylation of the CpG island in the promoter region and EZH2-mediated histone H3 lysine 27 trimethylation. Moreover, LINC00261 exerted its biological function by binding to miR-222-3p to activate the HIPK2/ERK/c-myc pathway. In addition, LINC00261 could also reduce c-myc expression by sequestering IGF2BP1. Our study suggests that LINC00261 functions as a tumor suppressor in pancreatic cancer and identifies novel epigenetic and posttranscriptional regulatory mechanisms of LINC00261, which contribute to the targeted therapy of pancreatic cancer.

## Introduction

Pancreatic cancer is one of the most malignant cancers of the digestive system and is characterized by late diagnosis and poor prognosis, with a 5-year survival rate of <6% [1]. Chemotherapy is the only option for most patients despite widespread chemoresistance. Recently, precision medicine and targeted therapy have become a topic of increasing interest [2]. However, a limited number of functional targets and their underlying mechanisms in pancreatic cancer have been identified. Therefore, novel therapeutic targets are urgently needed for the treatment of pancreatic cancer.

Long noncoding RNA (lncRNA), as a class of RNA transcripts with high abundance but limited or no protein-coding capacity, is gaining increasing interest in the scientific community [3]. Extensive studies have reported that lncRNAs are involved in various biological processes, including the immune response, inflammation, and cancer progression [4–6]. Epigenetic change is one of the most common modifications controlling the expression and tissue specificity of lncRNAs [7, 8]. LINC00261 has been extensively reported as a tumor suppressor involved in cell proliferation, migration, invasion, and chemoresistance in multiple cancers, such as lung cancer, gastric cancer, and colorectal cancer [9–11]. However, little is known about its function and underlying mechanism in pancreatic cancer. MiR-222-3p has been mostly reported as an oncogene involved in cancer progression [12–14], while few studies have indicated otherwise [15, 16]. However, its role in pancreatic cancer remains unclear. Homeodomain-interacting protein kinase 2 (HIPK2) is a well-studied tumor suppressor controlling a wide spectrum of biological functions, including hypoxia, cell proliferation, invasion, apoptosis, and the DNA damage response [17–19]. Recent research has demonstrated that HIPK2 attenuates glycolysis in pancreatic cancer by regulating the ERK/c-myc axis [20]. N^6^-methyladenosine is a newly discovered modification responsible for mRNA splicing, stability, translation, and subcellular localization, which mostly relies on m^6^A readers, such as IGF2BP1 [21, 22]. Interestingly, c-myc is one of the targets regulated by IGF2BP1 [23].

In this study, using bioinformatic analysis followed by biological validation, we revealed epigenetic modification resulting in aberrant expression of LINC00261 in pancreatic cancer. Moreover, we identified LINC00261 as a tumor suppressor with clinical significance that functions by sponging miR-222-3p to activate the HIPK2/ERK pathway and sequestering IGF2BP1 to attenuate the c-myc-mediated glycolytic process, which, in turn, inhibited the proliferation of pancreatic cancer cells.

## Results

### LINC00261 is significantly inhibited in pancreatic cancer tissues and cell lines and is associated with advanced pathological stage and poor prognosis

**Fig. 1:**
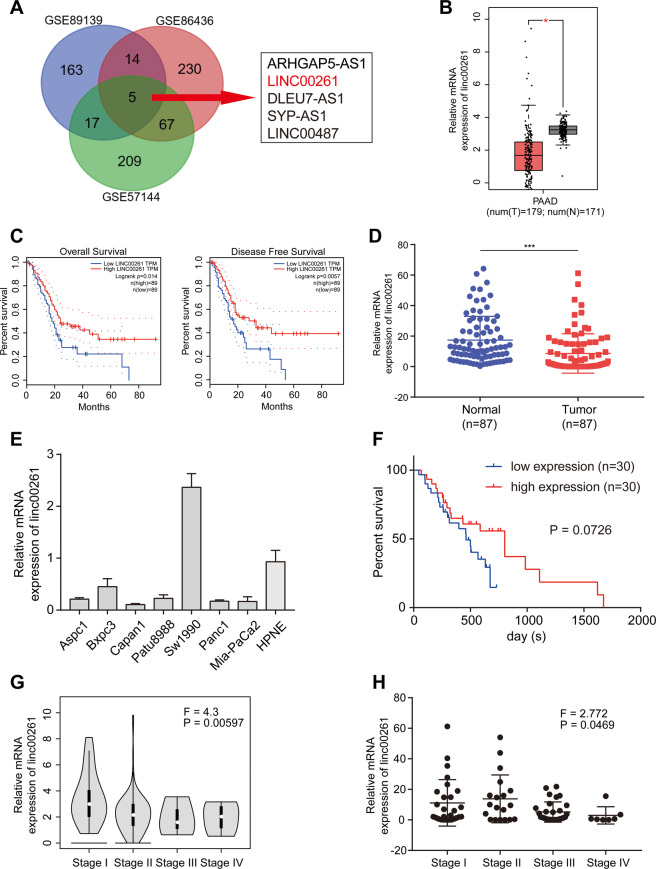
LINC00261 is significantly downregulated in pancreatic cancer and has clinical significance. **A** Venn diagram showing differentially expressed lncRNAs and five overlapping lncRNAs from GSE89139, GSE86436, and GSE57144. **B** Differential and **C** prognostic analyses of LINC00261 in the GEPIA database using data from TCGA. The expression of LINC00261 in **D** 87 pairs of pancreatic cancer and adjacent normal tissues and **E** pancreatic cancer cell lines. **F** Prognostic analysis of LINC00261 using survival data of 60 patients from our center. The expression of LINC00261 in patients divided by cancer stages using expression data from **G** TCGA database (*n* = 179) and **H** our center (*n* = 87). The data are presented as the mean ± SD of three independent experiments. *P < 0.05; ***P < 0.001.

We first performed differential analysis using expression data from GSE89139, GSE86436, and GSE57144 (Fig. S1) and identified five lncRNAs (ARHGAP5-AS1, LINC00261, DLEU7-AS1, SYP-AS1, and LINC00487) that were differentially expressed in all three Gene Expression Omnibus (GEO) data sets (Fig. 1A). Subsequently, to verify the results above and to investigate their prognostic potential, we obtained and analyzed expression and survival data from Gene Expression Profiling Interactive Analysis (GEPIA), which is based on The Cancer Genome Atlas (TCGA) database. It was found that although ARHGAP5-AS1 was upregulated in pancreatic cancer, it was not associated with patient survival (Fig. S2A). In addition, the DLEU7-AS1 and LINC00487 data showed no differential expression or prognostic value (Fig. S2B, C), and data on SYP-AS1 could not be found in TCGA database (Fig. S2D). However, LINC00261 was significantly downregulated and associated with both overall survival and disease-free survival (Fig. 1B, C). Therefore, we believe that LINC00261 is a potential functional lncRNA in pancreatic cancer. Using 87 pairs of patient samples from our center, we further confirmed that the expression of LINC00261 was suppressed in pancreatic cancer tissues (Fig. 1D). The results of quantitative real-time PCR (qRT-PCR) using cell lines showed that, except for Sw1990 cells, LINC00261 was significantly inhibited in pancreatic cancer cell lines compared with normal ductal pancreatic cells (Fig. 1E). In addition, we performed prognostic analysis using the survival data of 60 patients from our center and found that high expression of LINC00261 was correlated with better prognosis (Fig. 1F). Interestingly, we found that LINC00261 was inversely correlated with pathological stage or T stage using clinicopathological data from both TCGA database and our center (Tables 1 and 2 and Fig. 1G, H). All evidence suggests that LINC00261 could be a potential functional lncRNA in the progression of pancreatic cancer.

**Table. 1:** The correlation between LINC00261 expression and clinicopathological features of patients with pancreatic cancer from TCGA database.

Characteristics	*n* = 157	Expression of linc00261		*P* value
		Low expression	High expression	
Gender				
Male	86 (54.8%)	40 (46.5%)	46 (53.5%)	0.294
Female	71 (45.2%)	39 (54.9%)	32 (45.1%)	
Age (years)				
≥60	111 (70.7%)	55 (49.5%)	56 (50.5%)	0.765
<60	46 (29.3%)	24 (52.2%)	22 (47.8%)	
Pathologic stage				
I	18 (11.5%)	5 (27.8%)	13 (72.2%)	0.034*
II	132 (84.1%)	77 (58.3%)	55 (41.7%)	
III + IV	7 (4.5%)	5 (71.4%)	2 (28.6%)	
T stage				
T1 + T2	29 (18.5%)	11 (37.9%)	18 (62.1%)	0.103
T3 + T4	128 (81.5%)	70 (54.7%)	58 (45.3%)	
N stage				
N0	46 (29.3%)	22 (47.8%)	24 (52.2%)	0.801
N1	65 (41.4%)	32 (49.2%)	33 (50.8%)	
N2	46 (29.3%)	25 (54.3%)	21 (45.7%)	
M stage				
M0	76 (48.4%)	41 (53.9%)	35 (46.1%)	0.378
M1 + Mx	81 (51.6%)	38 (46.9%)	43 (53.1%)	
R stage				
R0	102 (65.0%)	46 (45.1%)	56 (54.9%)	0.075
R1 + R2	55 (35.0%)	33 (60.0%)	22 (40.0%)	

*TCGA* The Cancer Genome Atlas.

*P < 0.05 was considered to denote statistical significance.

**Table. 2:** The correlation between LINC00261 expression and clinicopathological features of patients with pancreatic cancer from our own center.

Characteristics	*n* = 87	Expression of linc00261		*P* value
		Low expression	High expression	
Gender				
Male	35 (40.2%)	18 (51.4%)	17 (48.6%)	0.630
Female	52 (59.8%)	28 (53.8%)	24 (46.2%)	
Age (years)				
≥60	55 (63.2%)	29 (52.7%)	26 (47.3%)	0.810
<60	32 (36.8%)	17 (53.1%)	15 (46.9%)	
Pathologic stage				
I	19 (21.8%)	6 (31.6%)	13 (68.4%)	0.012*
II	31 (35.6%)	16 (51.6%)	15 (48.4%)	
III + IV	37 (42.6%)	25 (67.6%)	12 (32.4%)	
T stage				
T1 + T2	32 (36.8%)	11 (34.4%)	21 (65.6%)	0.006*
T3 + T4	55 (63.2%)	35 (63.6%)	20 (36.4%)	
N stage				
N0	33 (37.9%)	13 (39.4%)	20 (60.6%)	0.161
N1	42 (48.3%)	23 (54.8%)	19 (45.2%)	
N2	12 (13.8%)	8 (66.7%)	4 (33.3%)	
M stage				
M0	76 (87.4%)	39 (51.3%)	37 (48.7%)	0.810
M1	11 (12.6%)	5 (45.5%)	6 (54.5%)	

*P < 0.05 was considered to denote statistical significance.

### Overexpression of LINC00261 suppressed glycolysis and proliferation of pancreatic cancer cells

**Fig. 2:**
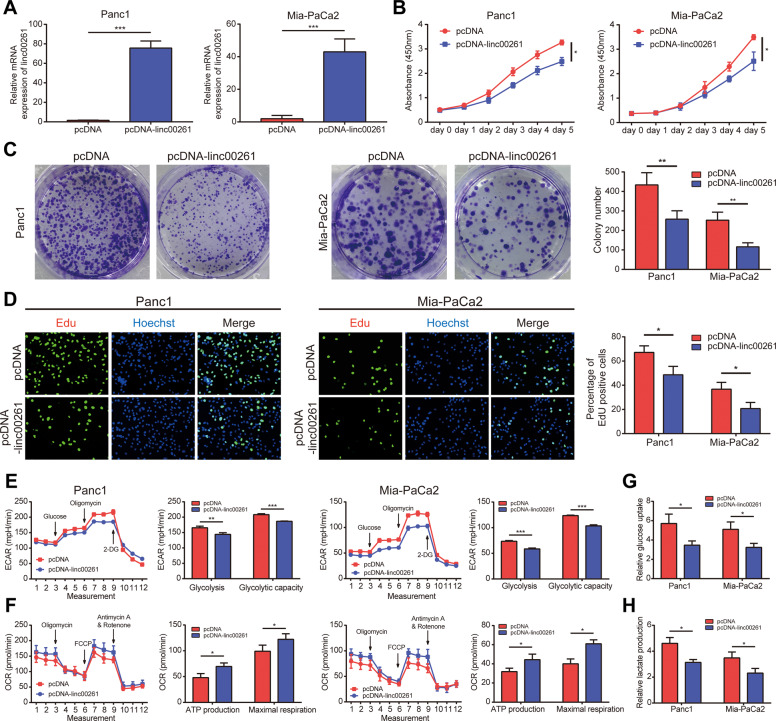
Overexpression of LINC00261 suppresses glycolysis and proliferation of pancreatic cancer cells in vitro. **A** Overexpression efficiency of LINC00261 in the pancreatic cancer cell lines Panc1 and Mia-PaCa2. Cell proliferation capacity of pancreatic cancer was measured by **B** CCK8 assay, **C** colony formation assay, and **D** EdU assay using Panc1 and Mia-PaCa2 cells. The glycolysis levels of Panc1 and Mia-PaCa2 cells were evaluated by **E** ECAR and **F** OCR, which were detected using a seahorse analyzer, and by **G** glucose uptake and **H** lactate production. The data are presented as the mean ± SD of three independent experiments. *P < 0.05; **P < 0.01; ***P < 0.001.

**Fig. 3:**
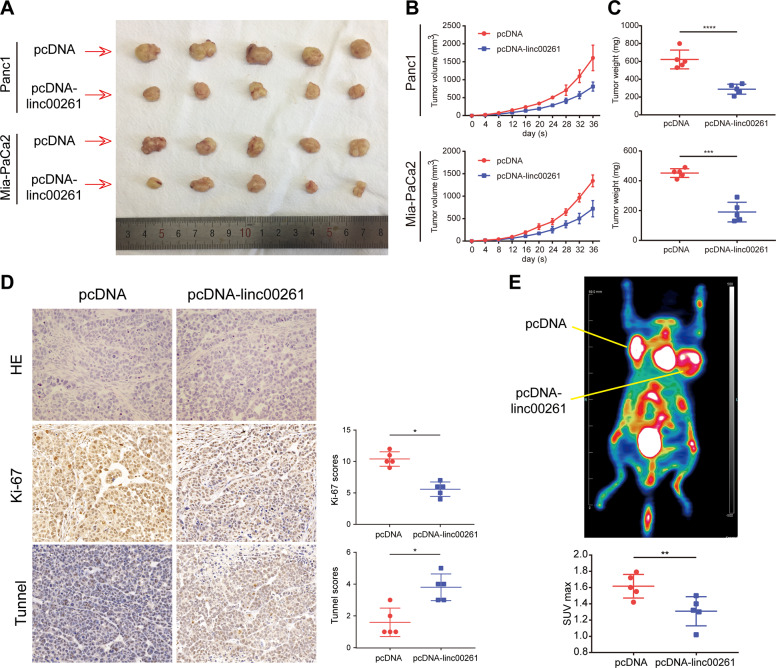
Overexpression of LINC00261 suppresses pancreatic tumor growth and metabolic levels in vivo. **A** Tumors were generated in nude mice using Panc1 and Mia-PaCa2 cells expressing LINC00261 or empty vector. **B** The tumor volume was calculated every 4 days from the initial injection. **C** The tumor weight was measured after resection. **D** IHC analysis using sliced tumor tissue derived from Panc1 cells incubated with Ki-67 and TUNEL antibodies. **E** Pet-CT imaging detecting the metabolic level of pancreatic tumors derived from Panc1 cells. *P < 0.05; **P < 0.01; ***P < 0.001; ****P < 0.0001.

Through correlation analysis using expression data from TCGA, we identified and selected the top 100 genes with the highest correlation coefficient. Then, we performed GSEA, and the results showed that they were mostly enriched in cancer-associated hallmark traits, such as glycolysis, MYC targets, epithelial–mesenchymal transition, and the P53 pathway (Fig. S3). Based on these results, we performed a CCK8 assay, colony formation assay, and EdU assay to measure the effect of LINC00261 on the proliferation capacity of pancreatic cancer cells. Panc1 and Mia-PaCa2 cells with low LINC00261 expression were selected as cell models that stably overexpressed LINC00261. The overexpression efficiency was detected by qRT-PCR (Fig. 2A). We found that forced expression of LINC00261 significantly inhibited pancreatic cancer proliferation (Fig. 2B–D). In addition, LINC00261 overexpression induced cell apoptosis and cell cycle arrest (Fig. S4). Moreover, using a seahorse analyzer, we found that LINC00261 overexpression could reduce the extracellular acidification rate (ECAR) and oxygen consumption rate (OCR) (Fig. 2E, F), as well as glucose consumption and lactate production (Fig. 2G, H), suggesting that LINC00261 could reduce glycolysis in pancreatic cancer. In vivo analysis further confirmed the results above. Overexpression of LINC00261 significantly suppressed tumor growth (Fig. 3A–C). Immunohistochemistry (IHC) analysis using tumor tissue resected from nude mice showed that tumors with high expression of LINC00261 exhibited lower Ki-67 expression and higher TUNEL staining (Fig. 3D). Moreover, PET-CT scanning showed that the overexpression of LINC00261 inhibited glucose metabolism in pancreatic cancer tissue (Fig. 3E). Interestingly, through in vitro analysis, we found that LINC00261 had no effect on the migration ability of pancreatic cancer cells (Fig. S5).

### Downregulation of LINC00261 in pancreatic cancer was caused by epigenetic modification

**Fig. 4:**
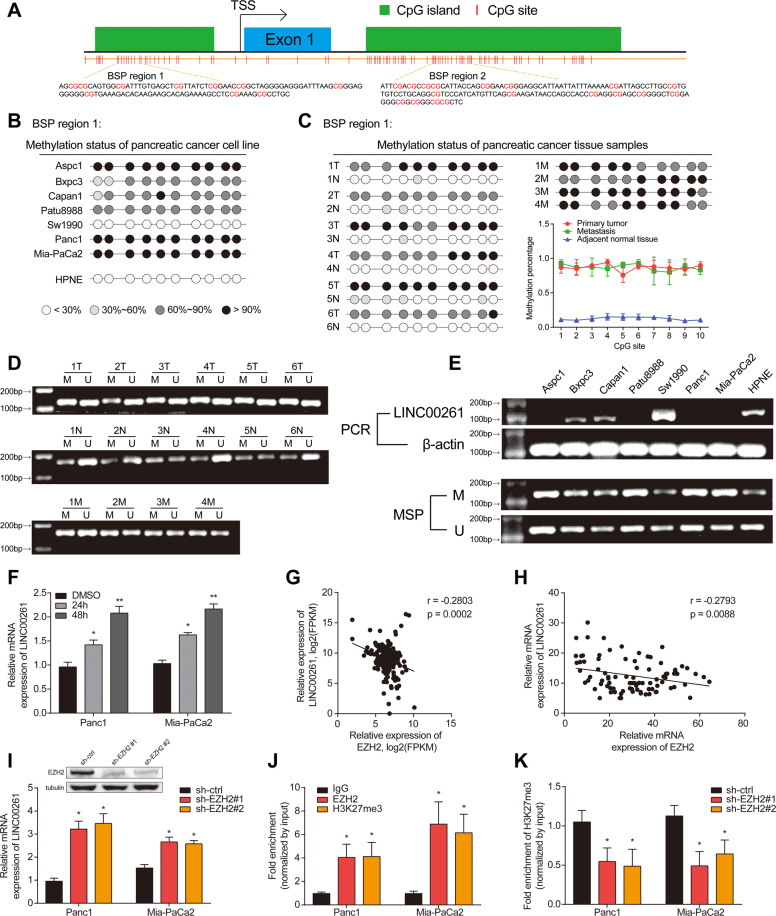
The downregulation of LINC00261 in pancreatic cancer is caused by epigenetic modification. **A** A diagram illustrating primer design for BSP analysis of the promoter region of LINC00261. Methylation level in BSP region 1 using DNA samples from **B** a pancreatic cancer cell line and normal pancreatic ductal epithelial cells and from **C** primary pancreatic cancer tissue, adjacent normal tissue, and metastatic tissue. **D** MSP analysis using DNA samples from six pairs of primary pancreatic cancer tissue and adjacent normal tissue and four cases of metastasis. **E** LINC00261 expression in pancreatic cancer cell lines and normal pancreatic ductal epithelial cells by qRT-PCR and the corresponding methylation level by MSP. **F** The expression of LINC00261 in Panc1 and Mia-PaCa2 cells after treatment with 5-azacytidine (DNA methyltransferase inhibitor) for 24 and 48 h, respectively. The correlation analysis between EZH2 and LINC00261 using data from **G** TCGA database and **H** our center. **I** The expression of LINC00261 in Panc1 and Mia-PaCa2 cells after knockdown of EZH2. **J** Fold enrichment of EZH2 and H3K27me3 in the promoter region of LINC00261 detected by ChIP assay. **K** The enrichment fold of H3K27me3 in the promoter region of LINC00261 after knockdown of EZH2. The data are presented as the mean ± SD of three independent experiments. *P < 0.05; **P < 0.01.

To investigate the mechanism mediating the downregulation of LINC00261 in pancreatic cancer, we hypothesized that DNA methylation could be involved [24]. Then, we used UCSC genome browser to predict the CpG islands in the promoter region of LINC00261. Next, we chose two regions (bisulfite sequencing PCR (BSP) regions 1 and 2) for BSP analysis (Fig. 4A). The results showed that the CpG islands in BSP region 1 and region 2 were hypermethylated in pancreatic cancer cell lines and tissues (Figs. 4B, C and S6). In addition, methylation-specific PCR (MSP) analysis further validated these results, showing that pancreatic cancer tissues had high methylation levels compared with normal tissue (Fig. 4D), and the expression of LINC00261 was inversely correlated with the methylation levels in the promoter region (Fig. 4E). After treatment with azacytidine (an inhibitor of DNA methyltransferases), the expression of LINC00261 was significantly increased (Fig. 4F). Apart from DNA methylation, EZH2-mediated H3K27 trimethylation is also a widely existing mechanism leading to silenced DNA transcription [25–27]. Therefore, we investigated the relationship between EZH2 and LINC00261. Through correlation analysis using data from TCGA and our center, we found that the expression of EZH2 was inversely associated with LINC00261 (Fig. 4G, H). In addition, silencing EZH2 promoted the expression of LINC00261 (Fig. 4I). According to the prediction by the online website hTFtarget based on chromatin immunoprecipitation (ChIP)-seq data, we performed ChIP analysis and found that EZH2 and H3K27me3 were significantly enriched in the promoter region of LINC00261 (Fig. 4J), and reduced EZH2 could inhibit the enrichment of H3K27me3 (Fig. 4K). The results above suggested that the downregulation of LINC00261 was caused by DNA hypermethylation and EZH2-mediated H3K27 trimethylation.

### LINC00261 regulated the HIPK2/ERK/c-myc axis by sponging miR-222-3p

**Fig. 5:**
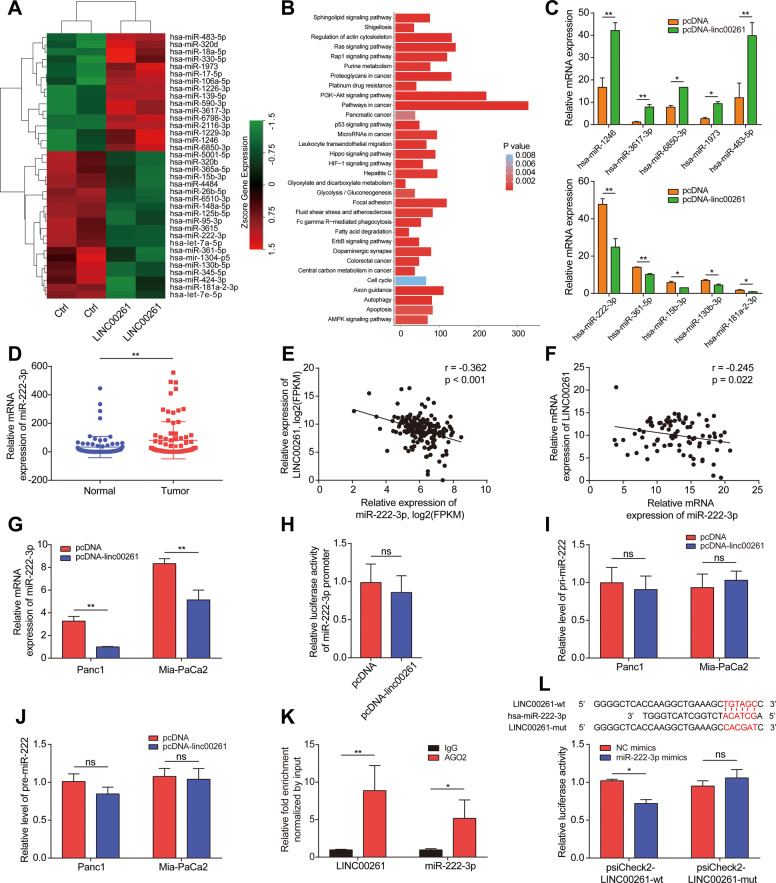
LINC00261 physically interacts with miR-222-3p. **A** Heatmap of differentially expressed miRNAs from miRNA sequencing data in Panc1 cells transfected with empty control or overexpressing LINC00261. **B** KEGG enrichment analysis using differentially expressed miRNAs. **C** Validation of the top 5 upregulated and downregulated miRNAs by LINC00261 with the most dramatic fold change in Panc1 cells. **D** The expression of miR-222-3p in patient samples from our center. The correlation analysis between LINC00261 and miR-222-3p using expression data from **E** TCGA database and **F** our center. **G** The expression of miR-222-3p in Panc1 and Mia-PaCa2 cells transfected with empty control or overexpressing LINC00261. **H** Promoter luciferase activity of miR-222-3p in HEK-293T cells transfected with empty control or overexpressing LINC00261. The expression change of **I** pri-miR-222 and **J** pre-miR-222 in Panc1 and Mia-PaCa2 cells transfected with empty control or overexpressing LINC00261. **K** RIP assay showing the fold enrichment of LINC00261 and miR-222-3p using AGO2 and IgG antibodies in Panc1 cells. **L** Luciferase activity in HEK-293T cells cotransfected with LINC00261 wild-type or mutant sequence and miR-222-3p mimics. The data are presented as the mean ± SD of three independent experiments. *P < 0.05; **P < 0.01. ns no significance.

**Fig. 6:**
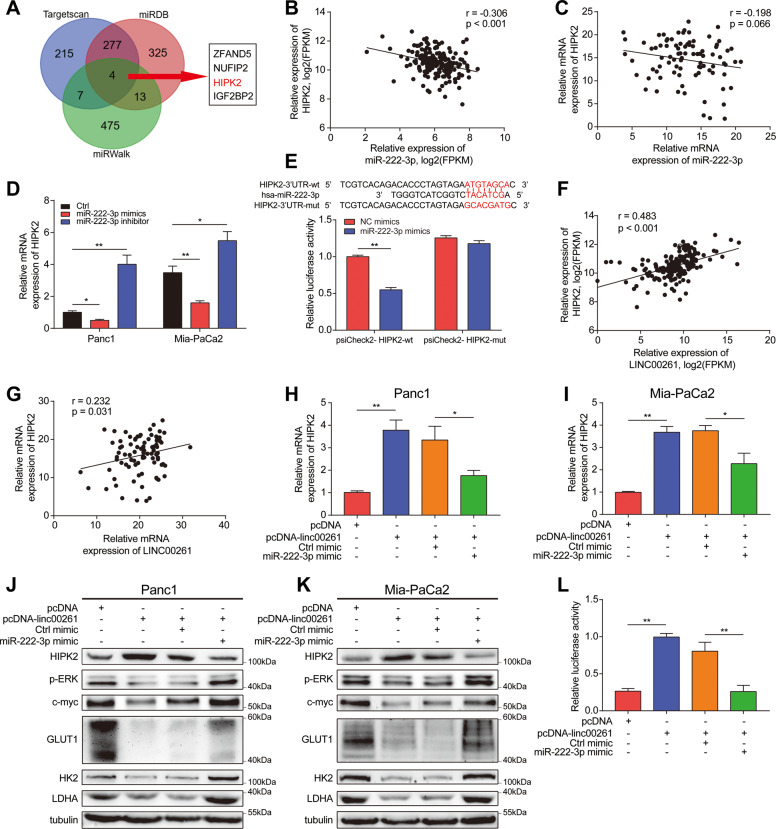
MiR-222-3p directly binds to the 3′UTR of HIPK2 and regulates the HIPK2/ERK/c-myc axis. **A** Predicted target genes of miR-222-3p using TargetScan, miRDB, and miRWalk and the four overlapping target genes. Correlation analysis between miR-222-3p and HIPK2 using data from **B** TCGA database and **C** our center. **D** The expression of HIPK2 in Panc1 and Mia-PaCa2 cells transfected with empty control or miR-222-3p mimics or inhibitor. **E** Luciferase activity in HEK-293T cells cotransfected with the HIPK2 wild-type or mutant sequence and miR-222-3p mimics. Correlation analysis between LINC00261 and HIPK2 using data from **F** TCGA database and **G** our center. The expression of HIPK2 in **H** Panc1 and **I** Mia-PaCa2 cells cotransfected with the LINC00261 overexpression vector and miR-222-3p mimics. The protein levels of the HIPK2/ERK/c-myc axis and c-myc target genes in **J** Panc1 and **K** Mia-PaCa2 cells. **L** Luciferase activity in HEK-293T cells cotransfected with LINC00261 overexpression vector and miR-222-3p mimics. The data are presented as the mean ± SD of three independent experiments. *P < 0.05; **P < 0.01.

To reveal the molecular mechanism through which LINC00261 exerts its tumor suppressive function, we first determined the subcellular location of LINC00261. Therefore, we first used the online prediction tool lncLocator. The results showed that LINC00261 was mainly distributed in the cytoplasm (Fig. S7A), which was then verified by nuclear and cytoplasmic separation and fluorescence in situ hybridization (FISH) assays. The results again indicated that the majority of LINC00261 was distributed in the cytoplasm (Fig. S7B, C). The competing endogenous RNA (ceRNA) mechanism is one of the most common mechanisms by which lncRNAs in the cytoplasm exert their functions [28]. Therefore, we hypothesized that LINC00261 could act as a ceRNA to bind with miRNAs. Then, we performed miRNA sequencing to screen for differentially expressed miRNAs between the empty vector group and LINC00261 overexpression group (Fig. 5A). The results of enrichment analysis using differentially expressed miRNAs showed that they were mainly enriched in pathways in cancer (Fig. 5B). Next, we validated the top 5 upregulated and top 5 downregulated miRNAs by qRT-PCR (Fig. 5C) and selected the most downregulated miRNA, miR-222-3p, as a downstream target. Using patient samples from TCGA database and our center, we found that miR-222-3p was significantly upregulated in pancreatic cancer tissues and was inversely correlated with LINC00261 (Fig. 5D–F). Overexpression of LINC00261 reduced the expression of miR-222-3p (Fig. 5G). To eliminate the possibility that LINC00261 is involved in the transcriptional regulation of miRNA via pri-miRNA or pre-miRNA synthesis, we detected the effect of LINC00261 on the promoter region and the expression of the pri-miRNA or pre-miRNA of miR-222-3p, and we found that LINC00261 had no effect on promoter activity or pri-miRNA or pre-miRNA expression (Fig. 5H–J). Moreover, by RNA immunoprecipitation (RIP) and dual-luciferase reporter assays, we found that LINC00261 could directly bind to miR-222-3p (Fig. 5K, L). Subsequently, we predicted the target genes of miR-222-3p using three online prediction websites and found four common target genes (ZFAND5, NUFIP2, HIPK2, and IGF2BP2) (Fig. 6A). Using patient samples, we found that while ZFAND5, NUFIP2, and IGF2BP2 were positively related to miR-222-3p, HIPK2 was inversely associated with miR-222-3p (Figs. S8A–C and 6B, C). The same results were obtained using pancreatic cell lines (Fig. 6D). Interestingly, previous research has established that HIPK2 can inhibit glycolysis and proliferation of pancreatic cancer by attenuating ERK/c-myc axis activity [20]. Further luciferase reporter assays showed that miR-222-3p could directly bind to the 3′UTR of HIPK2 (Fig. 6E). Therefore, we believe that HIPK2 is a functional target gene of miR-222-3p. In addition, LINC00261 was positively correlated with HIPK2 (Fig. 6F, G). Overexpression of miR-222-3p inhibited LINC00261-induced HIPK2 overexpression and HIPK2-mediated ERK/c-myc inactivation, as well as c-myc target genes (GULT1, HK2, and LDHA) (Figs. 6H, I and S8D–F). The above results indicated that LINC00261 could act as a ceRNA to release HIPK2 by sponging miR-222-3p. Functionally, miR-222-3p reversed the LINC00261 overexpression-induced decrease in cell proliferation and glycolysis and enhanced apoptosis and cell cycle arrest, similar to HIPK2 and miR-222-3p (Figs. S9 and S10). Collectively, the results above suggested that miR-222-3p was essential for the effect of LINC00261 on the HIPK2/ERK/c-myc axis.

### LINC00261 decreased c-myc mRNA stability by sequestering IGF2BP1

**Fig. 7:**
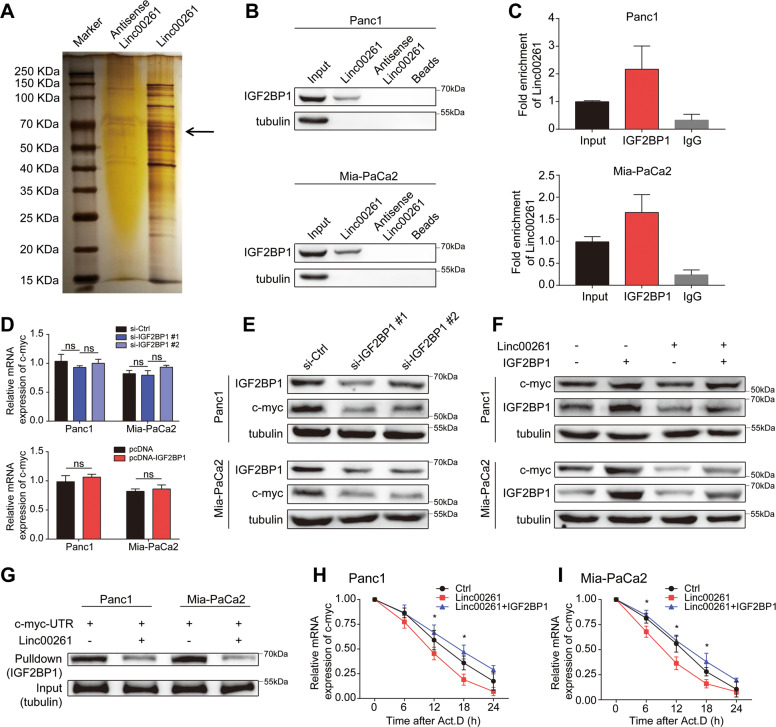
LINC00261 decreases c-myc mRNA stability by sequestering IGF2BP1. **A** Silver staining of LINC00261-associated proteins. One specific band (arrow) was excised and subjected to mass spectrometry. **B** Western blot of IGF2BP1 using protein samples enriched by biotinylated LINC00261, anti-LINC00261, and beads from Panc1 and Mia-PaCa2 cells. **C** Fold enrichment of LINC00261 in RNA samples precipitated by IGF2BP1 or IgG antibody in Panc1 and Mia-PaCa2 cells. The **D** mRNA and **E** protein levels of c-myc in Panc1 and Mia-PaCa2 cells with IGF2BP1 knockdown or overexpression. **F** The protein expression of c-myc in Panc1 and Mia-PaCa2 cells transfected with LINC00261 or cotransfected with LINC00261 and IGF2BP1. **G** Enrichment of IGF2BP1 measured using the biotinylated UTR region of c-myc in Panc1 and Mia-PaCa2 cells transfected with empty vector or vector with full-length LINC00261. The mRNA stability of c-myc in **H** Panc1 and **I** Mia-PaCa2 cells overexpressing LINC00261 or overexpressing LINC00261 and IGF2BP1 after treatment with actinomycin. The data are presented as the mean ± SD of three independent experiments. *P < 0.05. ns no significance.

To further discover the mechanisms through which LINC00261 exerts tumor suppressive functions, we performed an RNA pulldown assay followed by mass spectrometry and found that IGF2BP1 was a binding protein of LINC00261 (Fig. 7A). Further RNA pulldown and RIP assays confirmed this result (Fig. 7B, C). Recent evidence has suggested that IGF2BP1 is an RNA-binding protein that enhances mRNA stability and that c-myc is one of its target genes [23]. Using pancreatic cancer cell lines, we found that IGF2BP1 could not regulate the mRNA expression of c-myc but was positively related to the protein level of c-myc (Fig. 7D–F). Moreover, overexpression of LINC00261 could inhibit c-myc expression, while IGF2BP1 overexpression could rescue it (Fig. 7F). The results of the RNA pulldown assay using biotin-linked c-myc showed that the LINC00261 overexpression group contained far less IGF2BP1 than the control group (Fig. 7G). After treatment with actinomycin, the LINC00261 overexpression group exhibited a faster degradation rate of c-myc mRNA than the control group (Fig. 7H, I). The results above indicated that LINC00261 could regulate c-myc mRNA stability and therefore expression by sequestering IGF2BP1. Functionally, the LINC00261 overexpression-induced cell proliferation and glycolysis suppression, apoptosis acceleration, and cell cycle arrest could be reversed by IGF2BP1 overexpression (Fig. S11), suggesting that IGF2BP1 was necessary for LINC00261 to exert its tumor suppressive function.

**Fig. 8:**
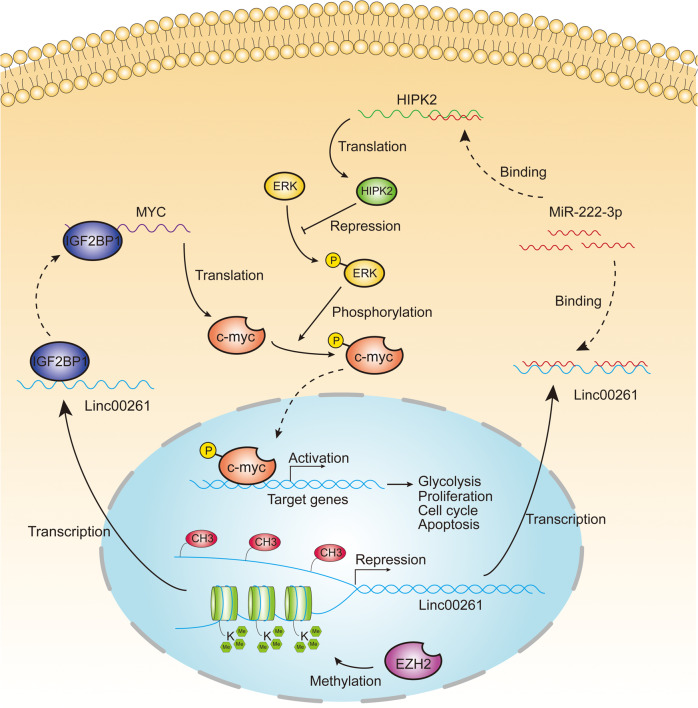
Proposed model demonstrating the epigenetic modification and functional significance of LINC00261 in the progression of pancreatic cancer. The expression of LINC00261 is repressed by DNA methylation and EZH2-mediated H3K27 trimethylation. LINC00261 exerts its tumor suppressive function by regulating miR-222-3p/HIPK2/ERK axis and sequestering IGF2BP1.

To investigate whether there is cross talk between the two axes, we determined the effect of miR-222-3p and HIPK2 on the expression of IGF2BP1, and vice versa. The results showed that miR-222-3p and HIPK2 had no effect on the mRNA or protein expression of IGF2BP1 (Fig. S12A–C). Similarly, IGF2BP1 overexpression or silencing could not regulate miR-222-3p or HIPK2 expression (Fig. S12D–H), suggesting that there was no cross-activation between the two axes. A proposed model demonstrating the epigenetic modification and regulatory pathways of LINC00261 is shown in Fig. 8.

## Discussion

Using clinical and expression data from TCGA database and our own center, we found that LINC00261 was significantly downregulated in pancreatic cancer tissues, correlated with advanced pathological stage, and had the potential to be a prognostic marker, which suggested that LINC00261 might be involved in the progression of pancreatic cancer. The results were partially in line with previously published articles [29, 30]. Although several studies have demonstrated that LINC00261 could function as a tumor suppressor in pancreatic cancer through its role in cell proliferation, migration, and invasion [31, 32], which is partially in accordance with our results, whether LINC00261 regulates glycolysis remains unclear. In our study, through in vitro and in vivo analyses, we first confirmed that LINC00261 could attenuate aerobic glycolysis in pancreatic cancer and revealed for the first time posttranscriptional regulatory mechanisms of LINC00261 acting as a ceRNA to sponge miR-222-3p and activate the HIPK2-mediated ERK/c-myc pathway and serving as a molecular decoy by sequestering IGF2BP1, which, in turn, decreased the mRNA stability of c-myc and thus reduced c-myc expression.

Previous research has indicated a controversial role of miR-222-3p in cancer progression involving cell proliferation, migration, apoptosis, and chemoresistance [12–16]. However, its role in pancreatic cancer has not been previously reported. In our study, we found that miR-222-3p was significantly upregulated in pancreatic cancer and acted as an oncogene to enhance glycolysis and proliferation by directly targeting HIPK2. A recent study discovered the suppressive effects of HIPK2 on proliferation and glycolysis in pancreatic cancer via inhibition of the ERK/c-myc axis [20], which was validated in our study and improved our understanding of the signal transduction and functional significance of LINC00261. More interestingly, by pulldown assay, we identified IGF2BP1 as a LINC00261 interacting protein. IGF2BP1 has been extensively reported as an oncogene involved in cell proliferation, invasion, stemness, and chemoresistance [33, 34] and has been recently established as a N^6^-methyladenosine reader responsible for mRNA stability [21, 22]. Several lncRNAs have been reported to regulate IGF2BP1 [35, 36]. However, for the first time, we revealed the relationship between LINC00261 and IGF2BP1, providing a novel posttranscriptional mechanism for IGF2BP1 regulation.

Apart from downstream pathways through which LINC00261 exerts its tumor suppressive functions, we also investigated the reason for its downregulation in pancreatic cancer, which has rarely been reported. BSP and MSP assays showed that the CpG islands in the promoter of LINC00261 exhibited significantly higher methylation levels in pancreatic cancer tissues and cell lines than in adjacent normal tissues and pancreatic ductal epithelial cells. In addition, ChIP analysis showed that EZH2 and H3K27 were enriched in the promoter region. The above results indicated that DNA methylation and EZH2-mediated H3K27 trimethylation were both responsible for aberrant expression of LINC00261 in pancreatic cancer, providing novel insight into the epigenetic modification of LINC00261 in the progression of pancreatic cancer.

Undoubtedly, there are some limitations in this study. First of all, compared with normal pancreatic epithelial cell, LINC00261 exhibits relatively low expression in pancreatic cancer cells, but not in SW1990, one of the aggressive cell pancreatic cancer cell lines. However, we investigated only on the cell lines with low expression of LINC00261, such as Panc1 and Mia-PaCa2. Therefore, the mechanisms leading to the high expression of LINC00261 in SW1990 need to be further elucidated. Second, the results of this whole study are based on commercially purchased cell lines, which cannot provide the most reliable in vitro and in vivo results. Therefore, primary pancreatic cancer cells extracted from patient tissues or PDX models should be applied for further validation.

In summary, we identified LINC00261 as a tumor suppressor with clinical significance. DNA methylation and EZH2-mediated H3K27 trimethylation were established for the first time to be responsible for the downregulation of LINC00261 in pancreatic cancer. LINC00261 attenuates c-myc-mediated aerobic glycolysis through two independent novel pathways: sponging miR-222-3p to increase the activity of the HIPK2/ERK/c-myc axis and sequestering IGF2BP1 to undermine c-myc mRNA stability. Overall, this study provides novel insight into epigenetic modification in the progression of pancreatic cancer and identifies a promising target for targeted therapy of pancreatic cancer.

## Methods

### Patient samples

Eighty-seven pairs of pancreatic cancer tissues and corresponding adjacent normal tissues were harvested from Ruijin Hospital Affiliated with Shanghai Jiaotong University School of Medicine. All patients enrolled met the following criteria: (1) pathologically diagnosed as pancreatic cancer; (2) with complete clinicopathological and follow-up data; (3) without preoperative chemotherapy. We obtained written informed consent from all patients involved, and our study protocol was approved by the ethics committee of Ruijin Hospital.

### Bioinformatic analysis

LncRNA expression arrays GSE8913, GSE86436, and GSE57144 were obtained from the GEO database. Differential analysis was performed using the GEO2R online tool. The expression and prognostic data of LINC00261 were also gathered and analyzed in GEPIA, which is based on TCGA and the Genotype-Tissue Expression project.

### Cell culture

Pancreatic cancer cell lines (Aspc1, Bxpc3, Capan1, Mia-PaCa2, Panc1, Sw1990, and Patu8988) and the human pancreatic ductal epithelial cell line were purchased from the Cell Bank of the Chinese Academy of Sciences. The cells were authenticated by STR, tested mycoplasma-negative, and were maintained in RPMI 1640, DMEM, and IMDM supplemented with 10% fetal bovine serum and antibiotics.

### Quantitative real-time PCR (qRT-PCR)

Total RNA from pancreatic tissues and cell lines was extracted using TRIzol Reagent (Invitrogen, CA, USA). The RNA from nuclear and cytoplasmic fractions was separated using the PARIS Kit. Reverse transcription was performed using HiScript III RT SuperMix (Vazyme, Nanjing, China). AceQ Universal SYBR qPCR Master Mix (Vazyme, China) was applied to perform qRT-PCR. The expression of β-tubulin was used as an internal mRNA control, and U6 was used as an internal miRNA control. The primer sequences are displayed in Table S1.

### Western blotting

RIPA buffer mixed with protease and phosphatase inhibitor cocktails was used to lyse the cell samples. The proteins were then separated by 10% SDS-PAGE and transferred onto PVDF membranes. Primary antibodies and appropriate secondary antibodies were used to detect the corresponding proteins. The antibodies used are listed in Table S2.

### Plasmid construction and cell transfection

Lipofectamine 2000 (Invitrogen, USA) was used to transfect the siRNAs, miRNA mimics/inhibitors, and plasmids into pancreatic cancer cell lines. Full-length complementary cDNAs of LINC00261, HIPK2, and IGF2BP1 were synthesized and inserted into the expression vector pcDNA3.1 (Bioegene, Shanghai, China). Panc1 and Mia-PaCa2 cells treated with puromycin for 48 h were chosen to establish stably overexpression and knockdown cell lines.

### Methylation-specific PCR (MSP) and bisulfite sequencing PCR (BSP)

Genomic DNA was extracted from pancreatic primary tissues, metastatic tissues, normal tissues, and cell lines with a QIAamp DNA Mini Kit (Qiagen, Germany). The purified DNA was then subjected to bisulfite modification with an EpiTect Bisulfite Kit (Qiagen, Germany) according to the manufacturer’s protocol. After MSP amplification, the DNA samples were separated by 2% agarose gel electrophoresis and visualized with GelRed (Vazyme, China). BSP analysis was performed using bisulfite-converted genomic DNA, and the sequencing library was prepared with the VAHTS Turbo DNA Library Prep Kit (Vazyme, China). The specific primers used for MSP and BSP are listed in Table S1.

### Fluorescence in situ hybridization (FISH) and immunohistochemistry (IHC) analysis

FISH analysis was performed using pancreatic cancer cell lines (Panc1 and Mia-PaCa2) to determine the subcellular location of LINC00261. The procedure was as previously described [37]. IHC analysis was performed using tumor tissues excised from tumor-bearing nude mice as previously described [37].

### Aerobic glycolysis analysis

The ECAR and OCR were considered as parameters to evaluate glycolysis stress and cell mitochondrial stress in pancreatic cancer cells using the Seahorse XF96 Glycolysis Analyzer (Seahorse Bioscience, MA, USA). In addition, glucose consumption, which was detected using the Glucose Uptake Cell-Based Assay Kit (Cayman, USA), and lactate production, which was measured using the Lactate Assay Kit-WST (Dojindo, Shanghai, China), were also quantified to detect the glycolysis level in pancreatic cancer cells.

### Cell proliferation assay

Stably transfected pancreatic cancer cells were plated in 96-well plates at equal numbers. Cell viability was measured using the CCK8 (Dojindo, China) assay. A colony formation assay (1000 cells/well in six-well plates) was also performed to detect the proliferation capacity of pancreatic cancer cells. In addition, a Cell-Light EdU DNA cell proliferation kit (RiboBio, Guangzhou, China) was used to detect the cell proliferation potential, following the manufacturer’s instructions.

### Flow cytometry analysis

Flow cytometry was applied to assess the cell cycle and apoptosis. For the cell cycle analysis, cells were harvested and fixed using 70% ethanol. After washing with PBS, cells were resuspended in 0.3 mL of PI/RNase Staining Buffer (BD Biosciences, San Jose, CA, USA), stained for 15 min at room temperature, and then subjected to flow cytometry analysis. For the apoptosis assay, the PE Annexin V Apoptosis Detection Kit I (BD Biosciences, USA) was used to detect the apoptosis rate. Adherent cells along with cells in the supernatant were collected. After washing with PBS, 3 µl of PE Annexin V and 5 µl of 7-AAD solution were added to each tube. Cells were incubated for 30 min in the dark and then subjected to flow cytometry analysis.

### Tumor xenograft assay

Nude BALB/c mice (male, 4–6 weeks old) were purchased from the Chinese Academy of Sciences (Shanghai, China) and were maintained in a specific pathogen-free facility. Panc1 and Mia-PaCa2 cells transfected with empty vector or LINC00261-expressing vector were injected subcutaneously into nude mice (5 × 10^6^ cells/site). Tumor volumes were measured every 4 days for 36 days from the first injection using the formula tumor volume (mm^3^) = 1/2 (*a* × *b*^2^), in which *a* represents the longest longitudinal diameter, and *b* is the longest transverse diameter. Panc1 cells transfected with empty vector or LINC00261-expressing vector were injected subcutaneously into nude mice (5 × 10^6^ cells/site). After 2 weeks, the mice were scanned by PET-CT to measure the metabolic activity of tumor tissues. All animal experimental procedures were performed in compliance with the institutional ethical requirements and were approved by the Shanghai Jiaotong University School of Medicine Committee for the Use and Care of Animals.

### Dual-luciferase reporter assays

A sequence containing the potential binding site of LINC00261 and the 3′UTR of HIPK2 and the corresponding mutant sequence were synthesized and cloned into the pGL3 luciferase reporter vector (Promega, Madison, WI, USA). HEK-293T cells were transfected with the plasmids described above. All luciferase activities were measured using the Dual-Luciferase Reporter Assay System (Promega, USA) and normalized to Renilla luciferase activity.

### RNA immunoprecipitation (RIP)

A Magna RIP RNA-Binding Protein Immunoprecipitation Kit (Millipore, Billerica, MA, USA) was used to perform RIP. Panc1 and Mia-PaCa2 cell lysates were incubated with RIP buffer containing magnetic beads conjugated with anti-Ago2 and anti-IGF2BP1 antibodies. Normal rabbit IgG was used as a negative control. The enriched RNA fragments were subjected to qRT-PCR.

### Chromatin immunoprecipitation (ChIP)

ChIP analysis was performed using the SimpleChIP Plus Sonication Chromatin IP Kit (CST, USA). Briefly, Panc1 and Mia-PaCa2 cells were cross-linked with 1% formaldehyde for 10 min and then quenched with glycine. The nuclear lysates were sonicated to generate appropriate DNA fragments, which were then incubated with EZH2 and H3K27me3 antibodies. Normal rabbit IgG was used as a negative control. The primers used for ChIP are listed in Table S1.

### RNA pulldown assay

Biotin-labeled LINC00261 and its antisense RNA were transcribed with Biotin RNA Labeling Mix (Roche Diagnostics, Indianapolis, IN, USA) and SP6/T7 RNA polymerase (Roche Diagnostics, USA). After purification, biotinylated RNAs were incubated with Panc1 cell lysates for 1 h at 4 °C. Streptavidin–agarose beads (Invitrogen, USA) were added to each tube for 1 h at room temperature. Finally, the enriched proteins were subjected to SDS-PAGE separation for mass spectrometry or western blot analysis.

### Statistical analysis

SPSS 23.0 and GraphPad Prism 7.0 were used to perform statistical analysis. The results were shown as mean ± SD. Two-sided Student’s *t* test was performed to compare the difference between two groups. Clinicopathological characteristics were analyzed by chi-square tests. Survival analysis was performed using the Kaplan–Meier method and log-rank tests. *P* < 0.05 was considered to indicate statistical significance. All experiments were performed in triplicate.

## Supplementary information

Supplemental table 1

Supplemental table 2

Supplemental figure 1

Supplemental figure 2

Supplemental figure 3

Supplemental figure 4

Supplemental figure 5

Supplemental figure 6

Supplemental figure 7

Supplemental figure 8

Supplemental figure 9

Supplemental figure 10

Supplemental figure 11

Supplemental figure 12

Supplemental figure legends
