# Supplementary material for: Epigenetic silencing of LncRNA LINC00261 promotes c-myc-mediated aerobic glycolysis by regulating miR-222-3p/HIPK2/ERK axis and sequestering IGF2BP1
Source: Oncogene. 2020 Oct 29;40(2):277–91. doi: 10.1038/s41388-020-01525-3 (PMC7808938; doi:10.1038/s41388-020-01525-3)
Supplement: Supplementary file 1 — Supplemental table 1 [file 41388_2020_1525_MOESM1_ESM.docx]

| **Table S1. Primers used for PCR** | | |
| --- | --- | --- |
| **Primers used for qRT-PCR** | | |
| LINC00261 | Forward | AAGACCAGCTCAACCATCGC |
|  | Reverse | TGCCATTTCCTGTGAATTGATGA |
| miR-222-3p | Forward | GCGGCAGTGCAATGATGAA |
|  | Reverse | CAGTGCAGGGTCCGAGGT |
| HIPK2 | Forward | CCCGTGTACGAAGGTATGGC |
|  | Reverse | AGTTGGAACTCGGCTCTATTTTC |
| IGF2BP1 | Forward | CTGAAGATCCTGGCCCATAA |
|  | Reverse | AAGGTCTTGCAACGAGGAGA |
| MYC | Forward | CGTCCTCGGATTCTCTGCTC |
|  | Reverse | GCTGGTGCATTTTCGGTTGT |
| **Primers used for MSP** | | |
| Methylated LINC00261 | Forward | TTTTTGGGTGAGTGTTAGGTGTAC |
|  | Reverse | AACCTACGAAACATAACGCGAT |
| Unmethylated LINC00261 | Forward | TTTTTGGGTGAGTGTTAGGTGTAT |
|  | Reverse | AAAACCTACAAAACATAACACAAT |
| **Primers used for BSP** | | |
| BSP region 1 | Forward | GCGGTCTGAGTTTGCAACTCTACG |
|  | Reverse | GCTCCCCTCCTCAGCGTCC |
| BSP region 2 | Forward | CGTCTGCCTGTTCGCCCGG |
|  | Reverse | CAGTTCACACCGGACCCG |
| **Primers used for CHIP** | | |
| LINC00261 | Forward | CCTTACAGGAAAGTGAACAG |
|  | Reverse | CATTAGCCATGCCATGGGT |
