# Supplementary material for: Epigenetic silencing of LncRNA LINC00261 promotes c-myc-mediated aerobic glycolysis by regulating miR-222-3p/HIPK2/ERK axis and sequestering IGF2BP1
Source: Oncogene. 2020 Oct 29;40(2):277–91. doi: 10.1038/s41388-020-01525-3 (PMC7808938; doi:10.1038/s41388-020-01525-3)
Supplement: Supplementary file 2 — Supplemental table 2 [file 41388_2020_1525_MOESM2_ESM.docx]

| **Table S2. Antibodies used for western blot or immunoprecipitation** | | | | |
| --- | --- | --- | --- | --- |
| **Antibody** | **Company** | **Cat. No.** | **Species** | **Dilution** |
| HIPK2 | Proteintech | 55408-1-AP | Rabbit | 1:1000 |
| p-ERK2 | Cell signaling | 4370 | Rabbit | 1:1000 |
| c-myc | Cell signaling | 18583 | Rabbit | 1:1000 |
| IGF2BP1 | Proteintech | 22803-1-AP | Rabbit | 1:1000 |
| EZH2 | Cell signaling | 5246 | Rabbit | 1:1000 |
| H3K27me3 | Abclonal | A2363 | Rabbit | 1:1000 |
| GLUT1 | Cell signaling | 12939 | Rabbit | 1:2000 |
| HK2 | Cell signaling | 2867 | Rabbit | 1:1000 |
| LDHA | Cell signaling | 3582 | Rabbit | 1:1000 |
| β-actin | Cell signaling | 4970 | Rabbit | 1:2000 |
| β-tubulin | Cell signaling | 2146 | Rabbit | 1:2000 |
