# Supplementary figures and images for: Epigenetic silencing of LncRNA LINC00261 promotes c-myc-mediated aerobic glycolysis by regulating miR-222-3p/HIPK2/ERK axis and sequestering IGF2BP1
Source: Oncogene. 2020 Oct 29;40(2):277–91. doi: 10.1038/s41388-020-01525-3 (PMC7808938; doi:10.1038/s41388-020-01525-3)

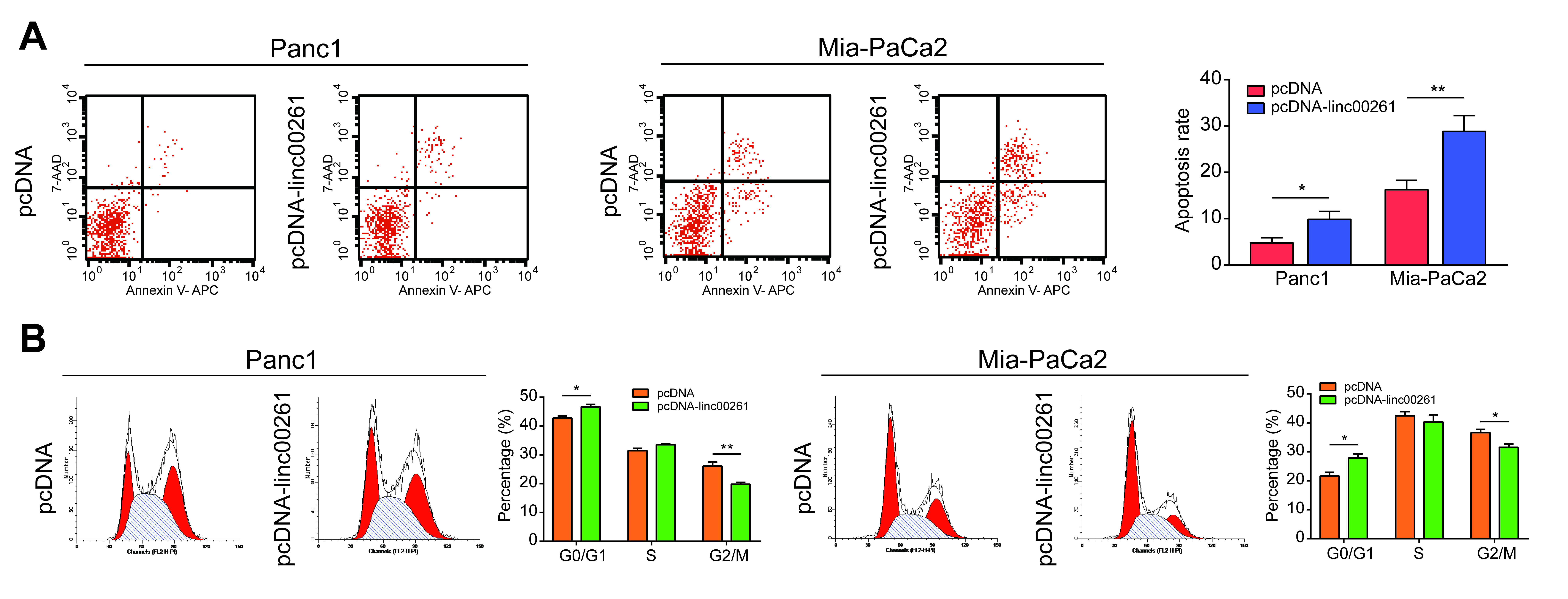

Supplement: Supplementary file 6 — Supplemental figure 4 [file 41388_2020_1525_MOESM6_ESM.tif]

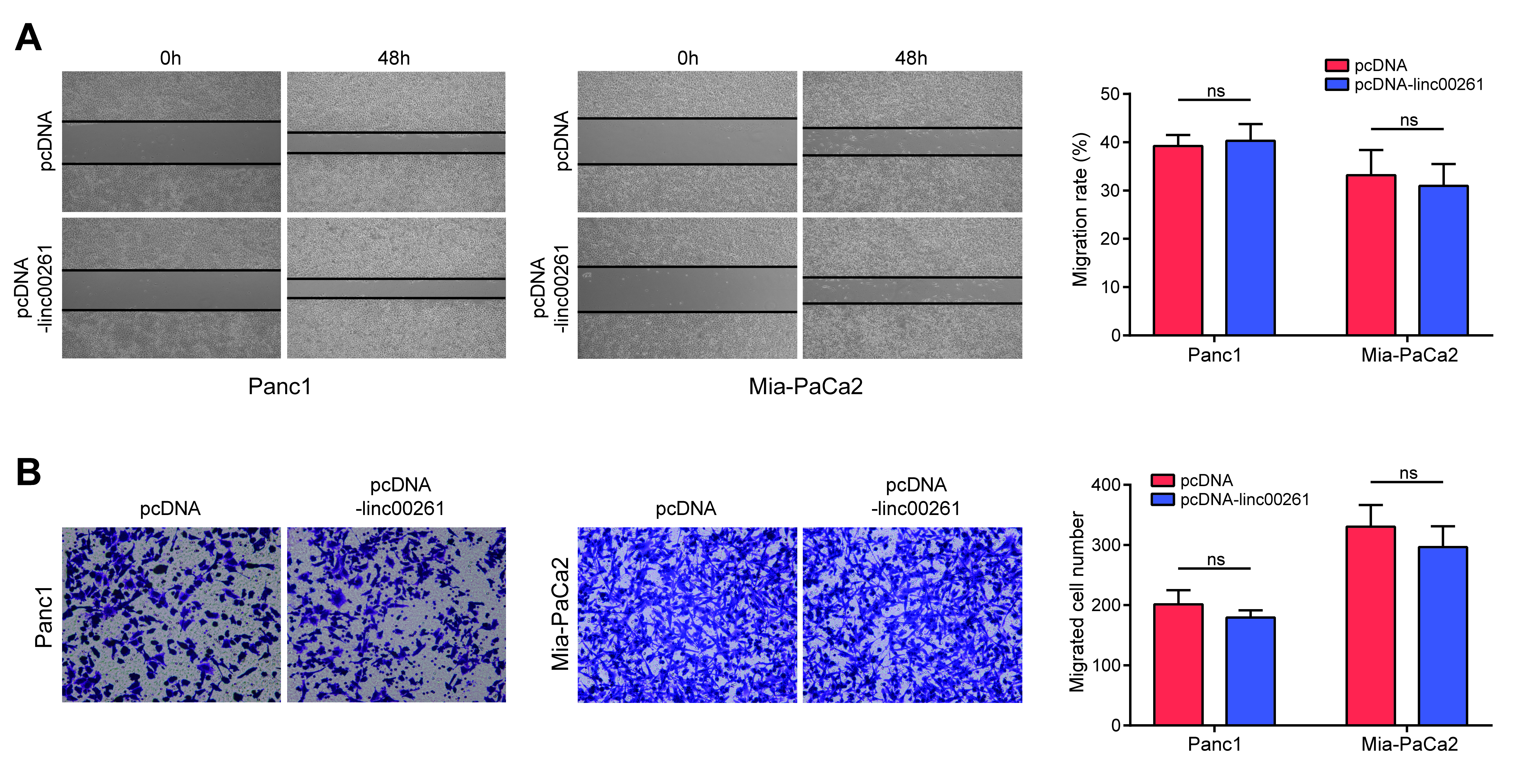

Supplement: Supplementary file 7 — Supplemental figure 5 [file 41388_2020_1525_MOESM7_ESM.tif]

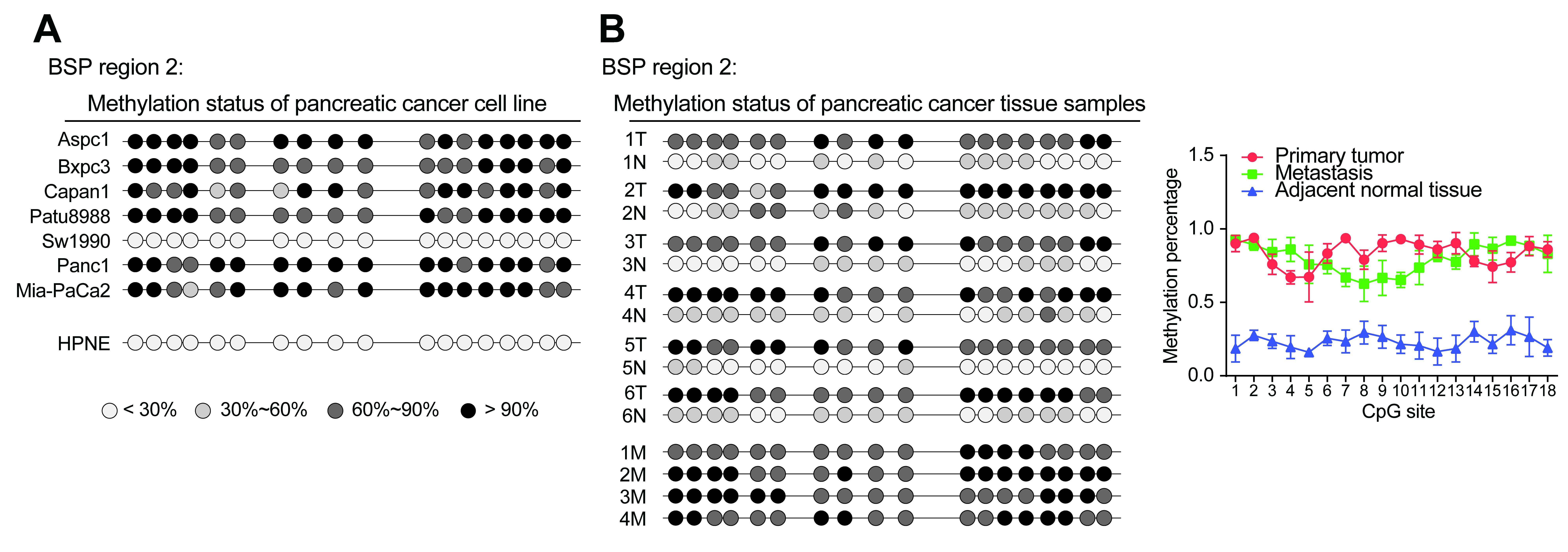

Supplement: Supplementary file 8 — Supplemental figure 6 [file 41388_2020_1525_MOESM8_ESM.tif]
