# Supplementary material for: Epigenetic silencing of LncRNA LINC00261 promotes c-myc-mediated aerobic glycolysis by regulating miR-222-3p/HIPK2/ERK axis and sequestering IGF2BP1
Source: Oncogene. 2020 Oct 29;40(2):277–91. doi: 10.1038/s41388-020-01525-3 (PMC7808938; doi:10.1038/s41388-020-01525-3)
Supplement: Supplementary file 15 — Supplemental figure legends [file 41388_2020_1525_MOESM15_ESM.pdf]

Figure S1. Differential analysis of GEO datasets. Heatmap showing the differentially expressed lncRNAs in (A) GSE89139, (B) GSE86436 and (C) GSE57144. Volcano plot illustrating the expression status of genes in (D) GSE89139, (E) GSE86436 and (F) GSE57144.

Figure S2. Differential and prognostic analysis in the GEPIA database. The expression difference between pancreatic cancer and normal tissue and prognostic analysis divided by the median expression level of (A) ARHGAP5-AS, (B) DLEU7-AS1, (C) LINC00487 and (D) SYP-AS1 in the GEPIA database, which is based on TCGA. Among them, the expression of SYP-AS1 could not be detected; therefore, prognostic analysis could not be performed. *$P < 0.05$

Figure S3. GSEA of LINC00261. Hallmarks of GSEA enrichment results using LINC00261 expression data from TCGA database

Figure S4. Overexpression of LINC00261 induced apoptosis and cell cycle arrest. (A) Apoptosis analysis using Panc1 and Mia-PaCa2 cells transfected with empty vector and LINC00261 expression vector. (B) Cell cycle analysis using Panc1 and Mia-PaCa2 cells transfected with empty vector and LINC00261 expression vector. *$P < 0.05$, **$P < 0.01$

Figure S5. LINC00261 had no effect on the migration ability of pancreatic cancer. (A) Wound healing assay and (B) Transwell assay using Panc1 and Mia-PaCa2 transfected with empty vector and LINC00261 expression vector. ns, not significant.

Figure S6. The promoter region of LINC00261 was hypermethylated in pancreatic cancer. (A) Methylation level in BSP region 2 using DNA samples from pancreatic cancer cell lines. (B) Methylation level in BSP region 2 using DNA samples from primary pancreatic cancer tissue, metastasis and adjacent

normal tissue.

Figure S7. LINC00261 was mainly distributed in the cytoplasm. (A) Predicted subcellular location of LINC00261 in the lncLocator online tool. (B) The expression of LINC00261 in the nucleus and cytoplasm detected by qRT-PCR in Panc1 and Mia-PaCa2 cells. GAPDH and ACTIN were used as positive controls in the cytoplasm. U6 was considered a positive control in the nucleus. (C) FISH analysis using Panc1 and Mia-PaCa2 cells showing the subcellular location of LINC00261. 18S was used as a positive control in the cytoplasm.

Figure S8. Correlation analysis. The correlation between miR-222-3p and (A) ZFAND5, (B) NUFIP2 and (C) IGF2BP2 using expression data from TCGA database. The correlation between LINC00261 and (D) GLUT1, (E) HK2 and (F) LDHA using expression data from TCGA database. $P < 0.05$ was considered to denote statistical significance.

Figure S9. MiR-222-3p and HIPK2 were necessary for LINC00261 to exert its tumor suppressive function. (A) Colony formation assay, (B) CCK8 assay and (C) EdU assay were performed to measure the proliferation capacity of pancreatic cancer using Panc1 and Mia-PaCa2 cells transfected with LINC00261 or cotransfected with LINC00261 and miR-222-3p mimic (another group was transfected with miR-222-3p or cotransfected with miR-222-3p and HIPK2). Glycolysis rate was measured by (D) ECAR and (E) OCR obtained by seahorse analyzer in Panc1 and Mia-PaCa2 cells with same treatment mentioned above. (F) Glucose uptake and (G) lactate production were also used to evaluate glycolysis in pancreatic cancer. $^*P < 0.05$, $^{**}P < 0.01$

Figure S10. Flow cytometry analysis detecting apoptosis and the cell cycle. (A) Apoptosis analysis

using Panc1 and Mia-PaCa2 cells transfected with LINC00261 or cotransfected with LINC00261 and miR-222-3p mimic (another group was transfected with miR-222-3p or cotransfected with miR-222-3p and HIPK2). (B) Cell cycle analysis using Panc1 and Mia-PaCa2 cells transfected with the same reagents mentioned above. *$P < 0.05$, **$P < 0.01$

Figure S11. IGF2BP1 was essential for LINC00261 to exert its tumor suppressive function. (A) Colony formation assay and (B) CCK8 assay were performed to measure the proliferation capacity of the pancreatic cancer Panc1 and Mia-PaCa2 cells transfected with LINC00261 or cotransfected with LINC00261 and IGF2BP1. (C) ECAR and (D) OCR were used to assess the glycolysis rate measured by a seahorse analyzer. (E) Glucose uptake and (F) lactate production were also used to evaluate glycolysis levels in pancreatic cancer. Flow cytometry analysis was used to detect (G) apoptosis and (H) the cell cycle in Panc1 and Mia-PaCa2 cells. *$P < 0.05$, **$P < 0.01$

Figure S12. IGF2BP1 had no cross talk with the miR-222-3p/HIPK2 axis. (A) IGF2BP1 mRNA levels in Panc1 and Mia-PaCa2 cells treated with miR-222-3p mimics or inhibitor. (B) IGF2BP1 mRNA levels in Panc1 and Mia-PaCa2 cells treated with empty vector or HIPK2 overexpression vector. (C) Protein level of IGF2BP1 in Panc1 and Mia-PaCa2 cells treated with miR-222-3p mimics or inhibitor and empty vector or HIPK2 overexpression vector. The expression of miR-222-3p in Panc1 and Mia-PaCa2 cells with (D) IGF2BP1 knockdown or (E) IGF2BP1 overexpression. The mRNA expression of HIPK2 in Panc1 and Mia-PaCa2 cells with (F) IGF2BP1 knockdown or (G) IGF2BP1 overexpression. (H) Protein expression of HIPK2 in Panc1 and Mia-PaCa2 cells with IGF2BP1 knockdown or IGF2BP1 overexpression. ns, not significant.
